# Mitochondrial genome variation in male LHON patients with the m.11778G > A mutation

**DOI:** 10.1007/s11011-020-00605-3

**Published:** 2020-08-01

**Authors:** Agnieszka Piotrowska-Nowak, Maciej R. Krawczyński, Ewa Kosior-Jarecka, Anna M. Ambroziak, Magdalena Korwin, Monika Ołdak, Katarzyna Tońska, Ewa Bartnik

**Affiliations:** 1 Institute of Genetics and Biotechnology, Faculty of Biology, University of Warsaw, 5a Pawińskiego Street, 02-106 Warsaw, Poland; 2 Department of Medical Genetics, Poznań University of Medical Sciences, 8 Rokietnicka Street, 60-806 Poznań, Poland; 3 Centers for Medical Genetics GENESIS, 4 Grudzieniec Street, 60-601 Poznań, Poland; 4 Department of Diagnostics and Microsurgery of Glaucoma, Medical University of Lublin, 1 Chmielna Street, 20-079 Lublin, Poland; 5 Faculty of Physics, University of Warsaw, 5 Pasteur Street, 02-093 Warsaw, Poland; 6 Department of Ophthalmology, Medical University of Warsaw, 13 Sierakowskiego Street, 03-709 Warsaw, Poland; 7 Department of Genetics, Institute of Physiology and Pathology of Hearing, 10 Mochnackiego Street, 02-042 Warsaw, Poland; 8 Department of Histology and Embryology, Center of Biostructure Research, Medical University of Warsaw, 5 Chałubińskiego Street, 02-004 Warsaw, Poland; 9 Institute of Biochemistry and Biophysics Polish Academy of Sciences, 5a Pawińskiego Street, 02-106 Warsaw, Poland

**Keywords:** Leber hereditary optic neuropathy (LHON), Mitochondrial DNA (mtDNA), M.11778G > A mutation, Next generation sequencing (NGS), Risk factors

## Abstract

Leber hereditary optic neuropathy (LHON) is a mitochondrial disorder with symptoms limited to a single tissue, optic nerve, resulting in vision loss. In the majority of cases it is caused by one of three point mutations in mitochondrial DNA (mtDNA) but their presence is not sufficient for disease development, since ~50% of men and ~10% women who carry them are affected. Thus additional modifying factors must exist. In this study, we use next generation sequencing to investigate the role of whole mtDNA variation in male Polish patients with LHON and m.11778G > A, the most frequent LHON mutation. We present a possible association between mtDNA haplogroup K and variants in its background, a combination of m.3480A > G, m.9055G > A, m.11299 T > C and m.14167C > T, and LHON mutation. These variants may have a negative effect on m.11778G > A increasing its penetrance and the risk of LHON in the Polish population. Surprisingly, we did not observe associations previously reported for m.11778G > A and LHON in European populations, particularly for haplogroup J as a risk factor, implying that mtDNA variation is much more complex. Our results indicate possible contribution of novel combination of mtDNA genetic factors to the LHON phenotype.

**Electronic supplementary material:**

The online version of this article (10.1007/s11011-020-00605-3) contains supplementary material, which is available to authorized users.

## Introduction

Maternally transmitted pathogenic variants of mitochondrial DNA (mtDNA) may lead to dysfunction of the oxidative phosphorylation system and the development of mitochondrial diseases, a heterogeneous group of usually multi-organ disorders that can occur at any age. Symptoms usually affect post-mitotic tissues with high energy demands, such as muscles and nerves (Greaves et al. 2012; Schon et al. 2012). The m.11778G > A point mutation was one of the first mtDNA pathogenic variants to be associated with human disease (Wallace et al. 1988). It was described in patients with hereditary predisposition to blindness, a disease known as Leber hereditary optic neuropathy (LHON) (Wallace et al. 1988).

LHON is one of the most peculiar mtDNA diseases. Although mitochondrial disorders are generally rare, LHON is the most common in this group (Chinnery et al. 2000; Kim et al. 2018). Sudden, painless and progressive loss of vision is the only symptom in the majority of cases. This clinical presentation exclusively limited to a single tissue results from retinal ganglion cell damage and loss, and characteristic degeneration of the optic nerve, however, the cause(s) for this phenomenon are still under study (Koilkonda and Guy 2011; Yu-Wai-Man et al. 2011). Over 90% of LHON cases are caused by one of three mtDNA point mutations, m.3460G > A, m.11778G > A or m.14484 T > C, in genes encoding subunits 1, 4 and 6, respectively, of respiratory chain complex I (Mackey et al. 1996; Tońska et al. 2010; Piotrowska et al. 2015; Caporali et al. 2017) and the m.11778G > A variant is the most frequent LHON mutation (accounting for 70–90% of all cases), associated with disease worldwide (Lott et al. 2013; Meyerson et al. 2015; Kim et al. 2018). Although necessary, their presence is not sufficient for disease development, implying that additional modifying factors, genetic, epigenetic and/or environmental, must exist (Yu-Wai-Man et al. 2011; Giordano et al. 2014; Piotrowska et al. 2015; Meyerson et al. 2015; Bianco et al. 2016; Caporali et al. 2017). Besides incomplete penetration, LHON also shows gender bias as symptoms affect ~50% of men and only ~10% of women who carry one of the three most common LHON mutations (Yu-Wai-Man et al. 2009; Tońska et al. 2010; Koilkonda and Guy 2011; Piotrowska et al. 2015; Meyerson et al. 2015).

One of the most frequently studied and well known modifying genetic factors of LHON are mtDNA haplogroups that are sets of specific common mitochondrial genetic variants segregating together and reflecting mtDNA evolutionary history. Increased risk of vision loss was observed when the m.14484 T > C mutation was present on the background of haplogroup J1, m.11778G > A on J1 and J2 and m.3460G > A on K, whereas haplogroup H had a protective effect in the case of m.11778G > A (Brown et al. 1997; Man 2004; Carelli et al. 2006; Hudson et al. 2007; Piotrowska et al. 2015; Meyerson et al. 2015; Caporali et al. 2017). These observations compellingly suggested that one or more common mtDNA variants defining these haplogroups act synergistically with primary LHON mutations to modify the risk of developing the disease.

The above-mentioned associations were found in European populations, however, studies on mtDNA variation show that there are significant differences between distinct populations in the frequency of individual mtDNA variants and haplogroups, highlighting the necessity for carefully designed, ethnically matched case-control studies. Thus extrapolation of results from one population to another might cause serious errors. With the advent of next-generation sequencing (NGS) methods, high-throughput analysis of whole genomes, including mitochondrial ones, was enabled, allowing for powerful and sophisticated screening for genetic modifiers of hereditary traits. In this study, we use NGS to investigate the mtDNA variation in male Polish LHON patients with the m.11778G > A mutation to confirm previously reported or to find novel mitochondrial genetic modifying factors. We found that haplogroup K and mtDNA variants defining it, m.3480A > G, m.9055G > A, m.11299 T > C and m.14167C > T, might increase the risk of LHON in male patients with the m.11778G > A mutation in the Polish population.

## Material

In this work only male cases were studied as men are primarily affected by this disorder. Since LHON shows incomplete penetration and gender bias, women could not be easily enrolled as they very rarely have the disease. The whole mtDNA analysis was performed in a total of 89 Polish male individuals. The patient group consisted of 47 unrelated men (mean age 28.3 ± 9.70) with diagnosed Leber Hereditary Optic Neuropathy and confirmed presence of the m.11778G > A mtDNA mutation. LHON samples included in this study were collected in the Medical University of Warsaw and the Centers for Medical Genetics GENESIS. The control group consisted of 42 unrelated men (mean age 71.0 ± 9.25) treated at the Department of Diagnostics and Microsurgery of Glaucoma, Medical University of Lublin, because of simple senile cataract. LHON (with the presence of any of three most common causal mutations in mtDNA), glaucoma and other intraocular pathologies were excluded. In all cases total DNA collected from peripheral blood was used. Mitochondrial sequence data of control individuals included in this study were extracted from datasets obtained previously in different study (Piotrowska-Nowak et al. 2018) and re-used for the analysis here.

## Methods

### mtDNA sequencing and analysis

High-throughput sequencing of mitochondrial genomes was performed as described previously (Piotrowska-Nowak et al. 2018, 2019). In brief, whole mtDNA was amplified using long-range PCR. PCR products were subsequently purified and quantified before proceeding to DNA library preparation for next generation sequencing using Illumina’s Nextera XT protocol and sequencing on MiSeq instrument. The NGS data were processed using CLC Genomics Workbench software (CLC bio, Qiagen). Bioinformatic analysis workflow consisted of quality control of sequencing reads, mapping to the human mtDNA reference sequence (rCRS, GenBank sequence NC_012920), variant detection, annotation and evaluation based on the strategy described in detail previously (Piotrowska-Nowak et al. 2018). mtDNA haplogroup assignment was performed for each subject using all variants identified in their mtDNA and the Mitomaster tool (Lott et al. 2013). The variant population frequency was obtained using a current human mtDNA sequence dataset deposited in GenBank and available from MITOMAP database (Lott et al. 2013). The population frequency cutoff value for rare mtDNA variants was set to ≤0.5%. Prediction of the impact of amino acid substitutions and analysis of patient’s sequence variant load was performed using a MutPred algorithm as described previously (Pienaar et al. 2017; Venter et al. 2017; Piotrowska-Nowak et al. 2019). Bioinformatic analysis of NGS data in search of large mtDNA deletions was performed using CLC Genomics Workbench InDels and Structural Variants tool and eKLIPse tool (Goudenège et al. 2018).

### Statistical analysis

The statistical analysis of mtDNA variation between LHON patients and control subjects was performed using IBM SPSS Statistics and GraphPad Prism software. Mitochondrial haplogroup distribution and frequency of individual mtDNA SNVs were compared using Fisher’s exact test together with odds ratio (OR) and 95% confidence intervals (95% CI) calculations of the strength of association. To assess the differences between rare (≤0.5%) and common (>0.5%) variant frequency, in the ratio of transitions to transversions and non-synonymous to synonymous variants in particular mtDNA regions Fisher’s exact test and OR with 95% CI were also applied. Independent *t*-tests were used to compare mean MutPred derived variant loads. The number of subjects having rare variants or variants with MutPred score above 0.5 was compared using Fisher’s exact test. The differences were considered statistically significant when the *p* value was <0.05.

## Results

### Proven pathogenic variants

In this study we investigated the mtDNA sequence variation in male LHON patients with the m.11778G > A mutation and control male subjects. The mitochondrial genome was covered by an average sequencing depth of 5141 ± 1661X. In all patients the m.11778G > A mutation was found in the homoplasmic state (i.e. in all mtDNA molecules), except for four men in whom high levels of heteroplasmy (i.e. mixture of mtDNA molecules with different sequence) was detected, 72%, 75%, 84% and 92% respectively (subject IDs: 9594, L7–2018, L12–2007 and 10911, respectively). No other proven pathogenic variants were found either in LHON patients or control individuals. However, three distinct mtDNA variants reported previously to associate with LHON (Fauser et al. 2002; Abu-Amero and Bosley 2006; Dai et al. 2018) were identified in three different patients and one control subject, in all in the homoplasmic state, and are shown in more detail in Table 1. All three variants define haplogroups on the background of which they were detected in patients (Oven and Kayser 2009). The presence of large deletions of mtDNA, that is above 1 kb, was also investigated. We did not observe any additional products in LR-PCR indicating the presence of mtDNA molecules with large deletions. Reliable rearrangements in mtDNA of studied participants were also not detected with bioinformatic analysis of NGS data.

**Table 1:** mtDNA variants reported to associate with LHON based on MITOMAP database

mtDNA variant	**Locus**	**Effect**	**GenBank frequency**	**Patient group (*n*** **= 47)**	**Control group (*n*** **= 42)**	**Status of association**	**Defined haplogroup /subject’s background**	**Subject ID**
m.3497C > T	*MT-ND1*	p.Ala64Val	0.35%	2%	0%	reported/ secondary	B4c1/B4c1b2	L7–2018
m.8836A > G	*MT-ATP6*	p.Met104Val	0.28%	2%	2%	reported	N1b/N1b1a2	9594 and KJ106
m.14831G > A	*MT-CYB*	p.Ala29Thr	0.20%	2%	0%	reported	L1c3b2, H13a1a1a, H50, B5b5, U8b1a2/ U8b1a2b	L39–2008

### mtDNA haplogroup distribution

Based on a full set of sequence variants detected with NGS, each subject’s mtDNA was assigned to the appropriate haplogroup. A total of 12 different mtDNA haplogroups were identified in the studied groups. Their observed frequencies in both cohorts are shown in Table 2. Comparison of mtDNA haplogroup distribution showed no statistically significant differences between men with LHON and control subjects. Only a borderline significant difference was noted for haplogroup K and its marginally higher than expected prevalence in LHON patients compared to controls (11% vs 0%, *p* = 0.057). For the individual mtDNA haplogroup details see Supplementary Table S1 in Online Resource 1.

**Table 2:** Prevalence of mtDNA haplogroups among LHON male patients and control individuals. Statistical analysis was performed using the Fischer’s exact test. Statistical significance was assumed at the level of *p* < 0.05

mtDNA haplogroup	Patient group (n = 47)	Control group (n = 42)	*p*	OR	95% CI
B	2%	0%	1.000	2.742 ^a^	0.109–69.144
H	32%	43%	0.380	0.625	0.263–1.486
HV	2%	2%	1.000	0.891	0.054–14.710
I	4%	2%	1.000	1.822	0.159–20.854
J	11%	17%	0.537	0.595	0.174–2.041
K	11%	0%	0.057	11.000 ^a^	0.590–205.205
N	4%	2%	1.000	1.822	0.159–20.854
T	6%	2%	0.619	2.795	0.279–27.961
U	19%	12%	0.396	1.753	0.537–5.722
V	2%	12%	0.096	0.161	0.018–1.438
W	4%	2%	1.000	1.822	0.159–20.854
X	2%	5%	0.600	0.435	0.038–4.976

^a^Haldane’s correction was applied to calculate OR when one of the cells contained zero values

### SNV association analysis

We also compared the frequency of individual variants in whole mtDNA between LHON patients and healthy individuals. A statistically significant difference was observed for five variants depicted in Table 3. All of them were found in the homoplasmic state and were more prevalent in the patient group and thus associated with increased risk of LHON in the studied men. Four out of five detected variants are markers of haplogroup U8b or haplogroup K which is phylogenetically derived from U8b (based on the mtDNA phylogenetic tree (Oven and Kayser 2009)). Moreover, those variants were identified together, exclusively on the background of the haplogroups they define, largely K, in the same six LHON subjects (except for one case of m.11299 T > C in a patient with haplogroup H). A m.73A > G change is a common variant present in many lineages of the human mtDNA tree (Oven and Kayser 2009; Lott et al. 2013), yet it was overrepresented in the LHON patient group in this study.

**Table 3:** List of mtDNA variants found to associate with LHON and m.11778G > A in this study. Statistical analysis was performed using the Fischer’s exact test. Statistical significance was assumed at the level of *p* < 0.05

mtDNA variant	**Locus**	**Region/Effect**	**Frequency [%]**		***p***	**OR**	**95% CI**	**Remarks**
			**Patient group (*n*** **= 47)**	**Control group (*n*** **= 42)**				
m.73A > G	*MT-CR*	non-coding	66%	43%	0.035	2.583	1.095–6.097	common throughout the human mtDNA tree
m.3480A > G	*MT-ND1*	p.Lys58=	13%	0%	0.028	13.313 ^a^	0.727–243.917	U8b’c marker
m.9055G > A	*MT-ATP6*	p.Ala177Thr	13%	0%	0.028	13.313 ^a^	0.727–243.917	U8b marker
m.11299 T > C	*MT-ND4*	p.Thr180=	13%	0%	0.028	13.313 ^a^	0.727–243.917	K marker
m.14167C > T	*MT-ND6*	p.Glu169=	13%	0%	0.028	13.313 ^a^	0.727–243.917	U8b marker

^a^Haldane’s correction was applied to calculate OR when one of the cells contained zero values

### Regional variant distribution

We have divided the mitochondrial genome into seven regions based on the function of the encoded products, as described previously (Piotrowska-Nowak et al. 2018): respiratory chain complex I, III and IV coding regions, ATP synthase coding region, rRNA and tRNA coding regions and non-coding region. For each of the studied groups in each region of mtDNA, as well as in the entire mtDNA, the number of transitions (T_S_) and transversions (T_V_) together with their ratio (T_S_/T_V_) was determined. The obtained results are presented in Table 4. No statistically significant differences in the ratio of transitions to transversions were noted for all categories tested.

**Table 4:** Number of transitions (T_S_), transversions (T_V_), and their ratio in particular mtDNA regions in male LHON patients and control subjects. Statistical analysis was performed using the Fischer’s exact test. Statistical significance was assumed at the level of *p* < 0.05. ‘Overall’ category represents calculations on the entire mtDNA variation and ‘overall unique’ – on the entire and unique (not iterated) mtDNA variation

mtDNA region	**Patient group**			**Control group**			***p***	**OR**	**95% CI**
	**Number of T**_**S**_	**Number of T**_**V**_	**T**_**S**_**/T**_**V**_	**Number of T**_**S**_	**Number of T**_**V**_	**T**_**S**_**/T**_**V**_			
Complex I	298	10	**29.80**	220	7	**31.43**	1.000	0.948	0.355–2.530
Complex III	126	10	**12.60**	86	10	**8.60**	0.479	1.465	0.585–3.671
Complex IV	86	2	**43.00**	72	2	**36.00**	1.000	1.194	0.164–8.693
ATP synthase	78	2	**39.00**	62	2	**31.00**	1.000	1.258	0.172–9.186
rRNA	188	0	**n/a**	143	1	**143.00**	0.434	3.941	0.159–97.450
tRNA	33	0	**n/a**	23	0	**n/a**	n/a	n/a	n/a
Non-coding	380	10	**38.00**	296	6	**49.33**	0.800	0.770	0.277–2.144
Overall	1189	34	**34.97**	902	28	**32.21**	0.795	1.086	0.653–1.803
Overall unique	303	20	**15.15**	286	18	**15.89**	1.000	0.953	0.494–1.839

**Fig. 1:**
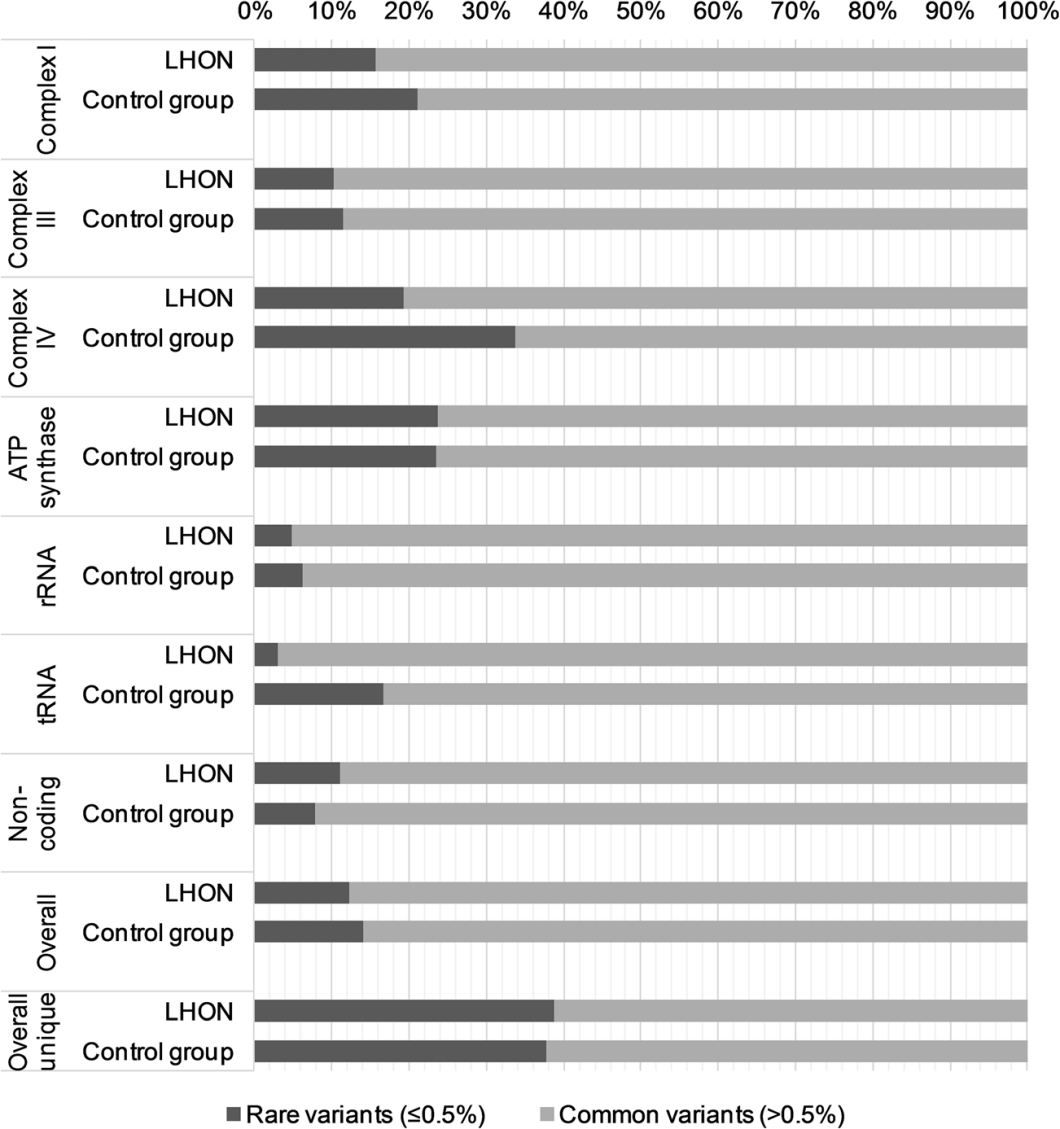
Frequency of rare and common variants in different regions of mtDNA in male LHON patients and control individuals

Subsequently we determined the number of rare (≤0.5% population frequency) and common (>0.5% population frequency) variants in each region of mtDNA. Their contribution in the sequence variation of particular mtDNA regions in both groups is shown in Fig. 1. Comparison of variant distribution showed a statistically significant difference in the frequency of rare variants in the region covering genes encoding subunits of respiratory chain complex IV (*p* = 0.048, Table 5). Specifically, rare variants in this region were more frequent in control individuals than in LHON patients (34% vs 19%). Healthy men were more likely to have at least one rare variant in this region compared to LHON patients (45% vs 30%), however, the difference was not large enough to be statistically significant (*p* = 0.187, OR = 0.514, 95% CI: 0.215–1.228). A large but not significant difference observed in the frequency of rare tRNA variants between both groups was associated with very few variants detected.

**Table 5:** Statistical analysis of the frequency of rare variants in particular mtDNA regions was performed using the Fischer’s exact test. Statistical significance was assumed at the level of *p* < 0.05. ‘Overall’ category represents calculations on the entire mtDNA variation and ‘overall unique’ – on the entire and unique (not iterated) mtDNA variation

mtDNA region	***p***	**OR**	**95% CI**
Complex I	0.111	0.688	0.442–1.072
Complex III	0.831	0.887	0.384–2.048
Complex IV	0.048	0.469	0.229–0.960
ATP synthase	1.000	1.017	0.469–2.207
rRNA	0.628	0.754	0.292–1.951
tRNA	0.151	0.156	0.016–1.499
Non-coding	0.136	1.459	0.917–2.323
Overall	0.216	0.854	0.671–1.087
Overall unique	0.812	1.041	0.763–1.421

We also determined the number of non-synonymous and synonymous variants in each protein coding region of mtDNA and compared their ratios between both groups. Statistical analysis did not show any significant differences between both groups (Table 6).

**Table 6:** Non-synonymous (NS) and synonymous (S) variant distribution and their ratios were determined in different mtDNA regions in LHON patients and control individuals. Statistical analysis was performed using the Fischer’s exact test. Statistical significance was assumed at the level of *p* < 0.05. ‘Overall’ category represents calculations on the entire mtDNA variation and ‘overall unique’ – on the entire and unique (not iterated) mtDNA variation

mtDNA region	**Patient group**			**Control group**			***p***	**OR**	**95% CI**
	**Number of NS**	**Number of S**	**NS/S**	**Number of NS**	**Number of S**	**NS/S**			
Complex I	59	249	0.24	53	174	0.30	0.240	0.778	0.512–1.182
Complex III	113	23	4.91	88	8	11.00	0.077	0.447	0.191–1.046
Complex IV	12	76	0.16	13	61	0.21	0.519	0.741	0.315–1.740
ATP synthase	61	18	3.39	55	9	6.11	0.204	0.555	0.230–1.336
Overall	245	366	0.67	209	252	0.83	0.092	0.807	0.632–1.031
Overall unique	55	126	0.44	60	117	0.51	0.499	0.851	0.546–1.327

To avoid false positive results caused by the presence of the m.11778G > A mutation, we excluded it from calculations of transition, rare and non-synonymous variants in complex I region and both ‘overall’ categories.

### MutPred variant load

**Fig. 2:**
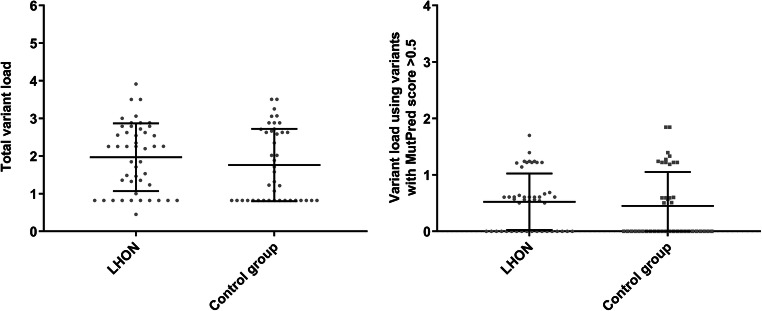
Distribution of variant loads based on all non-synonymous variants (left) and only non-synonymous variants with MutPred score above 0.5 threshold (right) in LHON men and control men. No significant differences were observed when comparing means in both groups with *t*-test for total variant load (patients 1.970 ± 0.901, controls 1.762 ± 0.961, *p* = 0.295) or MutPred score > 0.5 variant load (patients 0.525 ± 0.501, controls 0.452 ± 0.600, *p* = 0.531)

Variant load calculations were based on MutPred pathogenicity scores as described in detail elsewhere (Pienaar et al. 2017; Venter et al. 2017; Piotrowska-Nowak et al. 2019). In brief, MutPred scores for all non-synonymous variants detected in each subject’s mtDNA sequence were summed and the obtained value, total variant load, served as indicator of predicted mildly deleterious variants in participants. Additionally, as variants with high (>0.5) MutPred score are more likely to be deleterious to protein function, they were used to calculate second variant load, >0.5 threshold, as a clearer indication of a pathogenic load. m.11778G > A mutation was excluded from the calculations. Total and MutPred score > 0.5 variant load distributions in LHON patients and healthy subjects are shown in Fig. 2. No significant differences in the distribution of both variant load scores was observed. The number of subjects carrying variants with MutPred score > 0.5 did not differ significantly between the studied groups, however LHON patients were somewhat more likely to have at least one scored high pathogenicity variant (patients 62%, controls 43%, *p* = 0.091, OR = 2.148, 95% CI: 0.920–5.017). For the exact variant load values with the number of non-synonymous substitutions see Supplementary Table S1 in Online Resource 1.

## Discussion

LHON disease was first described almost 150 years ago (Leber 1871). Although our understanding of its pathogenesis has increased remarkably since then, there are still some aspects to be resolved, such as sex bias and incomplete penetration. In search of genetic modifying factors, we investigated whole mtDNA variation in male Polish LHON patients with m.11778G > A primary mutation.

Screening for the presence of other proven pathogenic variants in mtDNA, both point mutations and large deletions, brought negative results, implying that such deleterious changes do not contribute to disease development. Nevertheless, we identified three non-synonymous variants reported previously to associate with LHON (Fauser et al. 2002; Abu-Amero and Bosley 2006; Dai et al. 2018). However, their role in disease development in a synergistic mechanism, as an additional, secondary mutation, in three patients carrying them is debatable since their status was not yet confirmed with functional studies and thus their pathogenicity is unclear. Such variants accompanying primary LHON mutations are reported to be found also in unaffected control subjects, similarly as in one case in this study, and thus are suggested to be rather simple mtDNA variations with neutral effect, but it cannot be completely ruled out that their reported co-occurrence may confer phenotypic variability (Brown et al. 1997; Koilkonda and Guy 2011; Dai et al. 2018).

Haplogroup and SNP association analysis are commonly used methods applied in the search for mitochondrial genetic factors predisposing to or protecting from disease development. The modifying effect of mitochondrial genetic background on the penetrance of LHON mutations is well documented (Brown et al. 1997, 2000; Carelli et al. 2006; Hudson et al. 2007; Yu-Wai-Man et al. 2011; Piotrowska et al. 2015; Caporali et al. 2017). In this study mtDNA haplogroup K was overrepresented in LHON men compared to control individuals. Although the observed frequency difference was on the border of statistical significance, probably due to generally low frequency of haplogroup K in Polish population (about 4%, as reported in the latest large study by Jarczak et al. (2019)) and relatively small sample size in our study groups, subsequent SNP analysis revealed that four variants, markers of haplogroup U8b or K, which derives from U8b (Oven and Kayser 2009), associate with increased risk of LHON and have moderate to high effect size (OR > 2.5). These observations lead us to propose that haplogroup K and its characterizing variants, particularly specific combination of m.3480A > G, m.9055G > A, m.11299 T > C and m.14167C > T variants, may have a negative effect on m.11778G > A mutation and modulate LHON phenotype, its expression and/or penetrance, in male Polish patients. This is a novel finding since so far haplogroup J was commonly reported to associate with m.11778G > A and LHON in European population (Brown et al. 1997; Man 2004; Carelli et al. 2006; Hudson et al. 2007; Yu-Wai-Man et al. 2011; Meyerson et al. 2015). Moreover, so far haplogroup K was associated with increased risk of LHON for the m.3460G > A primary mutation in European patients (Hudson et al. 2007; Yu-Wai-Man et al. 2011; Meyerson et al. 2015), however, in previous studies this mutation was distributed randomly among mtDNA haplogroups (Torroni et al. 1997; Brown et al. 1997; Carelli et al. 2006). Discrepancies between those and our study might result from marked geographic variation of mtDNA haplogroups. However, it is conceivable that haplogroup K may have similar effects when associated with previously reported m.3460G > A and m.11778G > A described here in different populations. Further studies are needed to unravel this question.

Synergistic and deleterious effect of haplogroup K-related variants on the pathogenic potential of mtDNA mutation may come from subtle conformational changes shifting the assembly kinetics and stability of respiratory chain complexes, as was shown in LHON mutant cybrid cells belonging to different mtDNA haplogroups (Dudkina et al. 2005; Hudson et al. 2007; Pello et al. 2008; Yu-Wai-Man et al. 2011). Particularly interesting is the m.9055G > A variant which leads to an amino acid change in subunit 6 of ATP synthase and possibly may affect the efficiency of energy production in mitochondria. Although the remaining identified SNPs are synonymous variants, it is worth noting that all localize exclusively in genes encoding subunits of the respiratory chain complex I that is fundamental for proper functioning of the respiratory chain and that is affected by primary LHON mutations, including m.11778G > A. It has been recognized that silent sequence changes can impact the secondary structure or stability of mRNA and thus affect protein expression (Sauna and Kimchi-Sarfaty 2011, 2013) what can be true also in this case. Altogether, a partial complex I defect caused by the m.11778G > A mutation may be enhanced by further, normally subclinical, changes in the functioning of complex I and ATP synthase subunits associated with haplogroup K background. Such complex mtDNA variant interaction may thus impact LHON expression in Polish patients harboring the m.11778G > A mutation, increasing the probability for disease occurrence (Brown et al. 2002).

We also identified one common non-coding variant, m.73A > G, to be associated with increased risk of LHON in men with m.11778G > A in this study. It is an ancestral polymorphism present in multiple mtDNA lineages. It localizes in the main non-coding region of mtDNA referred to as the control region as it covers the main regulatory sequences associated with the replication and transcription of the mtDNA molecule (Anderson et al. 1981; Falkenberg et al. 2007). Therefore a non-coding control region variant, such as m.73A > G, will not directly affect the oxidative phosphorylation system altering energy or ROS production but, by changing a regulatory motif or being adjacent to one, it may impact the replication and transcription of the mitochondrial genome e.g. slightly influencing mtDNA copy number or gene expression (Suissa et al. 2009; Lott et al. 2013; Umbria et al. 2018). This together with other risk factors and m.11778G > A mutation may possibly modulate the LHON phenotype.

DNA sequence variants of a harmful nature are removed from the population through purifying selection. It is therefore assumed that pathogenic variants of mtDNA, even those mildly deleterious, will be rare and more frequently observed on the younger branches of the mtDNA phylogenetic tree (Elson et al. 2004; Pereira et al. 2011; Soares et al. 2013; Wei et al. 2017; Venter et al. 2017; Piotrowska-Nowak et al. 2019). According to the hypothesis “common disease - rare variant” (CDRV), a single rare variant may have mild deleterious effect, but such multiple changes may contribute to the development of the disease in a synergistic way by cumulative effect (Elson et al. 2006; Schork et al. 2009; Pienaar et al. 2017; Venter et al. 2017; Piotrowska-Nowak et al. 2019). Regarding mitochondria this seems particularly attractive because all genes in the mitochondrial genome encode products that are involved in the same basic biochemical process, oxidative phosphorylation. In order to verify this hypothesis, we compared the frequency of rare mtDNA variants between patients and controls in individual, arbitrarily determined, mtDNA regions. Analysis of sequence variation showed increased frequency of rare variants in the region covering genes encoding subunits of respiratory chain complex IV in healthy subjects when compared to LHON patients. This suggests that rare variants may also play a protective role in the development of disease, what was already reported previously (Singh et al. 2018).

Not all variants of low population frequency will actually be harmful, thus we also predicted the pathogenicity of variants in silico using the MutPred algorithm and compared variant cumulative impact between two groups. The usefulness and strength of the MutPred tool in estimating the harmfulness of non-synonymous mtDNA variants has been previously demonstrated (Pereira et al. 2011, 2012; Pienaar et al. 2017; Venter et al. 2017; Piotrowska-Nowak et al. 2019). In this study we did not observe any significant differences in MutPred variant load between patients and controls. However, this can be attributed to the small number of individuals in study groups and thus should be verified in larger cohorts.

The burden of possibly harmful variants was also investigated by analysis of transversions and non-synonymous variants, known to bear deleterious potential. We did not find any significant differences between the studied groups in any of the mtDNA regions tested what implies lack of association between transversion and non-synonymous variation load and risk of LHON associated with m.11778G > A in men in this study.

In summary, in this study we investigated the whole mtDNA variation in male patients with LHON and its association with the m.11778G > A mutation in the Polish population. Most interestingly, we present a possible association between mtDNA haplogroup K and variants in its background and m.11778G > A mutation. Although the presented data are preliminary with a limited sample size not allowing to make firm conclusions, our results indicate possible contribution of novel combination of mtDNA genetic factors to the LHON phenotype, e.g. by increasing the penetrance of the mutation in this mtDNA background. Surprisingly, we did not observe associations previously reported for m.11778G > A and LHON in European populations, particularly for haplogroup J as a risk factor, what implies that mtDNA variation is much more complex. Further investigation in larger cohorts are required to verify these important findings.

## Electronic supplementary material

ESM 1 (PDF 582 kb)

## Data Availability

Nucleotide sequence data reported are available in the GenBank database under the accession numbers MN176233-MN176279 and MG646186, MG646188, MG646195, MG646199-MG646204, MG646206, MG646210, MG646211, MG646213-MG646216, MG646221-MG646225, MG646230, MG646237, MG646239, MG646243, MG646246, MG646248, MG646251, MG646252, MG646254, MG646258, MG646259, MG646261, MG646262, MG646265, MG646266, MG646268, MG646269, MG646274, MG646276, MG646277, MG646278. The datasets generated and analyzed during the current study are available from the corresponding author on reasonable request.

## References

[CR1] Abu-Amero KK, Bosley TM (2006). Mitochondrial abnormalities in patients with LHON-like optic neuropathies. Invest Ophthalmol Vis Sci.

[CR2] Anderson S, Bankier AT, Barrell BG, de Bruijn MHL, Coulson AR, Drouin J, Eperon IC, Nierlich DP, Roe BA, Sanger F, Schreier PH, Smith AJH, Staden R, Young IG (1981). Sequence and organization of the human mitochondrial genome. Nature.

[CR3] Bianco A, Martínez-Romero I, Bisceglia L, D'Agruma L, Favia P, Ruiz-Pesini E, Guerriero S, Montoya J, Petruzzella V (2016). Mitochondrial DNA copy number differentiates the Leber’s hereditary optic neuropathy affected individuals from the unaffected mutation carriers. Brain.

[CR4] Brown MD, Starikovskaya E, Derbeneva O, Hosseini S, Allen JC, Mikhailovskaya IE, Sukernik RI, Wallace DC (2002). The role of mtDNA background in disease expression: a new primary LHON mutation associated with Western Eurasian haplogroup J. Hum Genet.

[CR5] Brown MD, Sun F, Wallace DC (1997). Clustering of Caucasian Leber hereditary optic neuropathy patients containing the 11778 or 14484 mutations on an mtDNA lineage. Am J Hum Genet.

[CR6] Brown MD, Trounce IA, Jun AS, Allen JC, Wallace DC (2000). Functional analysis of lymphoblast and Cybrid mitochondria containing the 3460, 11778, or 14484 Leber’s hereditary optic neuropathy mitochondrial DNA mutation. J Biol Chem.

[CR7] Caporali L, Maresca A, Capristo M, del Dotto V, Tagliavini F, Valentino ML, la Morgia C, Carelli V (2017). Incomplete penetrance in mitochondrial optic neuropathies. Mitochondrion.

[CR8] Carelli V, Achilli A, Valentino ML, Rengo C, Semino O, Pala M, Olivieri A, Mattiazzi M, Pallotti F, Carrara F, Zeviani M, Leuzzi V, Carducci C, Valle G, Simionati B, Mendieta L, Salomao S, Belfort R, Sadun AA, Torroni A (2006). Haplogroup effects and recombination of mitochondrial DNA: novel clues from the analysis of Leber hereditary optic neuropathy pedigrees. Am J Hum Genet.

[CR9] Chinnery PF, Johnson MA, Wardell TM, Singh-Kler R, Hayes C, Brown DT, Taylor RW, Bindoff LA, Turnbull DM (2000). The epidemiology of pathogenic mitochondrial DNA mutations. Ann Neurol.

[CR10] Dai Y, Wang C, Nie Z, Han J, Chen T, Zhao X, Ai C, Ji Y, Gao T, Jiang P (2018). Mutation analysis of Leber’s hereditary optic neuropathy using a multi-gene panel. Biomed Rep.

[CR11] Dudkina NV, Eubel H, Keegstra W, Boekema EJ, Braun HP (2005). Structure of a mitochondrial supercomplex formed by respiratory-chain complexes I and III. Proc Natl Acad Sci U S A.

[CR12] Elson JL, Herrnstadt C, Preston G, Thal L, Morris CM, Edwardson JA, Beal MF, Turnbull DM, Howell N (2006). Does the mitochondrial genome play a role in the etiology of Alzheimer’s disease?. Hum Genet.

[CR13] Elson JL, Turnbull DM, Howell N (2004). Comparative genomics and the evolution of human mitochondrial DNA: assessing the effects of selection. Am J Hum Genet.

[CR14] Falkenberg M, Larsson N-G, Gustafsson CM (2007). DNA replication and transcription in mammalian mitochondria. Annu Rev Biochem.

[CR15] Fauser S, Luberichs J, Besch D, Leo-Kottler B (2002). Sequence analysis of the complete mitochondrial genome in patients with Leber’s hereditary optic neuropathy lacking the three most common pathogenic DNA mutations. Biochem Biophys Res Commun.

[CR16] Giordano C, Iommarini L, Giordano L, Maresca A, Pisano A, Valentino ML, Caporali L, Liguori R, Deceglie S, Roberti M, Fanelli F, Fracasso F, Ross-Cisneros FN, D’Adamo P, Hudson G, Pyle A, Yu-Wai-Man P, Chinnery PF, Zeviani M, Salomao SR, Berezovsky A, Belfort R, Ventura DF, Moraes M, Moraes Filho M, Barboni P, Sadun F, de Negri A, Sadun AA, Tancredi A, Mancini M, d’Amati G, Loguercio Polosa P, Cantatore P, Carelli V (2014). Efficient mitochondrial biogenesis drives incomplete penetrance in Leber’s hereditary optic neuropathy. Brain.

[CR17] Goudenège D, Bris C, Hoffmann V, Desquiret-Dumas V, Jardel C, Rucheton B, Bannwarth S, Paquis-Flucklinger V, Lebre AS, Colin E, Amati-Bonneau P, Bonneau D, Reynier P, Lenaers G, Procaccio V (2018). eKLIPse: a sensitive tool for the detection and quantification of mitochondrial DNA deletions from next-generation sequencing data. Genetics in Medicine.

[CR18] Greaves LC, Reeve AK, Taylor RW, Turnbull DM (2012). Mitochondrial DNA and disease. J Pathol.

[CR19] Hudson G, Carelli V, Spruijt L, Gerards M, Mowbray C, Achilli A, Pyle A, Elson J, Howell N, la Morgia C, Valentino ML, Huoponen K, Savontaus ML, Nikoskelainen E, Sadun AA, Salomao SR, Belfort R, Griffiths P, Man PYW, de Coo RFM, Horvath R, Zeviani M, Smeets HJT, Torroni A, Chinnery PF (2007). Clinical expression of Leber hereditary optic neuropathy is affected by the mitochondrial DNA-haplogroup background. Am J Hum Genet.

[CR20] Jarczak J, Grochowalski Ł, Marciniak B, Lach J, Słomka M, Sobalska-Kwapis M, Lorkiewicz W, Pułaski Ł, Strapagiel D (2019). Mitochondrial DNA variability of the polish population. Eur J Hum Genet.

[CR21] Kim US, Jurkute N, Yu-Wai-Man P (2018). Leber Hereditary Optic Neuropathy—Light at the End of the Tunnel?. The Asia-Pacific Journal of Ophthalmology.

[CR22] Koilkonda RD, Guy J (2011). Leber’s hereditary optic neuropathy-gene therapy: from Benchtop to bedside. J Ophthalmol.

[CR23] Leber T, Royal College of Surgeons of England (1871) Ueber hereditäre und congenital-angelegte Sehnervenleiden. [Leipzig : s.n]

[CR24] Lott MT, Leipzig JN, Derbeneva O, Xie HM, Chalkia D, Sarmady M, Procaccio V, Wallace DC (2013). mtDNA variation and analysis using Mitomap and Mitomaster. Curr Protoc Bioinformatics.

[CR25] Mackey DA, Oostra RJ, Rosenberg T, Nikoskelainen E, Bronte-Stewart J, Poulton J, Harding AE, Govan G, Bolhuis PA, Norby S (1996). Primary pathogenic mtDNA mutations in multigeneration pedigrees with Leber hereditary optic neuropathy. Am J Hum Genet.

[CR26] Man PYW (2004). Mitochondrial DNA haplogroup distribution within Leber hereditary optic neuropathy pedigrees. J Med Genet.

[CR27] Meyerson C, Van Stavern G, McClelland C (2015). Leber hereditary optic neuropathy: current perspectives. Clin Ophthalmol.

[CR28] van Oven M, Kayser M (2009). Updated comprehensive phylogenetic tree of global human mitochondrial DNA variation. Hum Mutat.

[CR29] Pello R, Martín MA, Carelli V, Nijtmans LG, Achilli A, Pala M, Torroni A, Gómez-Durán A, Ruiz-Pesini E, Martinuzzi A, Smeitink JA, Arenas J, Ugalde C (2008). Mitochondrial DNA background modulates the assembly kinetics of OXPHOS complexes in a cellular model of mitochondrial disease. Hum Mol Genet.

[CR30] Pereira L, Soares P, Máximo V, Samuels DC (2012). Somatic mitochondrial DNA mutations in cancer escape purifying selection and high pathogenicity mutations lead to the oncocytic phenotype: pathogenicity analysis of reported somatic mtDNA mutations in tumors. BMC Cancer.

[CR31] Pereira L, Soares P, Radivojac P, Li B, Samuels DC (2011). Comparing phylogeny and the predicted pathogenicity of protein variations reveals equal purifying selection across the global human mtDNA diversity. Am J Hum Genet.

[CR32] Pienaar IS, Howell N, Elson JL (2017). MutPred mutational load analysis shows mildly deleterious mitochondrial DNA variants are not more prevalent in Alzheimer’s patients, but may be under-represented in healthy older individuals. Mitochondrion.

[CR33] Piotrowska A, Korwin M, Bartnik E, Tońska K (2015). Leber hereditary optic neuropathy - historical report in comparison with the current knowledge. Gene.

[CR34] Piotrowska-Nowak A, Elson JL, Sobczyk-Kopciol A et al (2019) New mtDNA association model, MutPred variant load, suggests individuals with multiple mildly deleterious mtDNA variants are more likely to suffer from atherosclerosis. Front genet 9. 10.3389/fgene.2018.00702 10.3389/fgene.2018.00702 PMC6332467 30671084

[CR35] Piotrowska-Nowak A, Kosior-Jarecka E, Schab A, Wrobel-Dudzinska D, Bartnik E, Zarnowski T, Tonska K (2018). Investigation of whole mitochondrial genome variation in normal tension glaucoma. Exp Eye Res.

[CR36] Sauna ZE, Kimchi-Sarfaty C (2011). Understanding the contribution of synonymous mutations to human disease. Nat Rev Genet.

[CR37] Sauna ZE, Kimchi-Sarfaty C (2013) Synonymous mutations as a cause of human genetic disease. In: John Wiley & Sons ltd (ed) eLS. John Wiley & Sons, ltd, Chichester, UK

[CR38] Schon EA, DiMauro S, Hirano M (2012). Human mitochondrial DNA: roles of inherited and somatic mutations. Nat Rev Genet.

[CR39] Schork NJ, Murray SS, Frazer KA, Topol EJ (2009). Common vs. rare allele hypotheses for complex diseases. Curr Opin Genet Dev.

[CR40] Singh LN, Crowston JG, Lopez Sanchez MIG, van Bergen NJ, Kearns LS, Hewitt AW, Yazar S, Mackey DA, Wallace DC, Trounce IA (2018). Mitochondrial DNA variation and disease susceptibility in primary open-angle Glaucoma. Invest Ophthalmol Vis Sci.

[CR41] Soares P, Abrantes D, Rito T, Thomson N, Radivojac P, Li B, Macaulay V, Samuels DC, Pereira L (2013). Evaluating purifying selection in the mitochondrial DNA of various mammalian species. PLoS One.

[CR42] Suissa S, Wang Z, Poole J, Wittkopp S, Feder J, Shutt TE, Wallace DC, Shadel GS, Mishmar D (2009). Ancient mtDNA genetic variants modulate mtDNA transcription and replication. PLoS Genet.

[CR43] Tońska K, Kodroń A, Bartnik E (2010). Genotype-phenotype correlations in Leber hereditary optic neuropathy. Biochim Biophys Acta.

[CR44] Torroni A, Petrozzi M, D’Urbano L (1997). Haplotype and phylogenetic analyses suggest that one European-specific mtDNA background plays a role in the expression of Leber hereditary optic neuropathy by increasing the penetrance of the primary mutations 11778 and 14484. Am J Hum Genet.

[CR45] Umbria M, Ramos A, Aluja MP, Santos C (2018) The role of control region mitochondrial DNA mutations in cardiovascular disease: stroke and myocardial infarction bioRxiv 382374. 10.1101/382374 10.1038/s41598-020-59631-x PMC7026394 32066781

[CR46] Venter M, Malan L, van Dyk E, Elson JL, van der Westhuizen FH (2017). Using MutPred derived mtDNA load scores to evaluate mtDNA variation in hypertension and diabetes in a two-population cohort: the SABPA study. J Genet Genomics.

[CR47] Wallace DC, Singh G, Lott MT, Hodge J, Schurr T, Lezza A, Elsas L, Nikoskelainen E (1988). Mitochondrial DNA mutation associated with Leber’s hereditary optic neuropathy. Science.

[CR48] Wei W, Gomez-Duran A, Hudson G, Chinnery PF (2017). Background sequence characteristics influence the occurrence and severity of disease-causing mtDNA mutations. PLoS Genet.

[CR49] Yu-Wai-Man P, Griffiths PG, Chinnery PF (2011). Mitochondrial optic neuropathies – disease mechanisms and therapeutic strategies. Prog Retin Eye Res.

[CR50] Yu-Wai-Man P, Griffiths PG, Hudson G, Chinnery PF (2009). Inherited mitochondrial optic neuropathies. J Med Genet.

