# Supplementary material for: Mitochondrial genome variation in male LHON patients with the m.11778G > A mutation
Source: Metab Brain Dis. 2020 Aug 1;35(8):1317–27. doi: 10.1007/s11011-020-00605-3 (PMC7584531; doi:10.1007/s11011-020-00605-3)
Supplement: Supplementary file 1 — (PDF 582 kb) [file 11011_2020_605_MOESM1_ESM.pdf]

Metabolic Brain Disease

Mitochondrial genome variation in male LHON patients with the m.11778G>A mutation

Agnieszka Piotrowska-Nowak [1,*], Maciej R. Krawczyński [2,3], Ewa Kosior-Jarecka [4], Anna M. Ambroziak [5], Magdalena Korwin [6], Monika Ołdak [7,8], Katarzyna Tońska [1], Ewa Bartnik [1,9]

[1] Institute of Genetics and Biotechnology, Faculty of Biology, University of Warsaw, 5a Pawińskiego Street, Warsaw, 02-106, Poland

[2] Department of Medical Genetics, Poznań University of Medical Sciences, 8 Rokietnicka Street, Poznań, 60-806, Poland

[3] Centers for Medical Genetics GENESIS, 4 Grudzieniec Street, Poznań, 60-601, Poland

[4] Department of Diagnostics and Microsurgery of Glaucoma, Medical University of Lublin, 1 Chmielna Street, Lublin, 20-079, Poland

[5] Faculty of Physics, University of Warsaw, 5 Pasteur Street, Warsaw, 02-093, Poland

[6] Department of Ophthalmology, Medical University of Warsaw, 13 Sierakowskiego Street, Warsaw, 03-709, Poland

[7] Department of Genetics, Institute of Physiology and Pathology of Hearing, 10 Mochnackiego Street, Warsaw, 02-042, Poland

[8] Department of Histology and Embryology, Center of Biostructure Research, Medical University of Warsaw, 5 Chałubińskiego Street, Warsaw, 02-004, Poland

[9] Institute of Biochemistry and Biophysics Polish Academy of Sciences, 5a Pawińskiego Street, Warsaw, 02-106, Poland

*Correspondence:

Agnieszka Piotrowska-Nowak

Email: apiotrowska@biol.uw.edu.pl

Supplementary Table S1 mtDNA haplogroup details, total variant loads and variant loads using only non-synonymous variants with MutPred score above 0.5 threshold calculations for all participants in the study

LHON male subject ID	Age in years	mtDNA haplogroup	Total variant load	Number of variants	Variant load using variants with MutPred score >0.5	Number of variants with MutPred score >0.5
**3595**	42	H10b	1.712	4	0.638	1
**4106**	33	H5a1a	0.821	2	0.000	0
**4806**	35	X2b+226	2.349	6	0.569	1
**5529**	42	U4a2a2	1.466	5	0.000	0
**6586**	33	U4c1	1.850	6	0.000	0
**9594**	35	N1b1a2	3.005	7	1.210	2
**9922**	42	J2a1a1a	3.506	9	1.396	2
**10911**	33	U5b1e1	1.533	5	0.000	0
**11266**	35	K1c1h	2.257	6	0.609	1
**11922**	42	U5a1a1	2.625	7	0.561	1
**13933**	33	H7a1	0.821	2	0.000	0
**14164**	35	U5a1a1	2.625	7	0.561	1
**15046**	42	K1c1	2.257	6	0.609	1
**15415**	33	H1b1a	1.361	3	0.540	1
**15591**	35	J1c2e1	2.883	8	1.220	2
**15745**	42	W5a2	2.719	7	0.505	1
**16159**	33	W6a	3.915	9	1.701	3
**16799**	35	H1	0.821	2	0.000	0
**17367**	42	J1c2e1	2.883	8	1.220	2
**17545**	33	I2e	2.261	6	0.606	1
**17791**	35	H1a	0.821	2	0.000	0
**194-L**	42	J1c2c2a	3.506	10	1.220	2
**456-L**	33	H1e	1.326	3	0.505	1
**L12-2007**	37	K2a5	2.548	6	0.609	1
**L12-2008**	23	T1a1b	2.793	7	1.239	2
**L12-2012**	n/a	H5a1	1.357	3	0.536	1
**L13-2006**	28	T1a1b	2.793	7	1.239	2
**L16-2008**	14	V13	0.821	2	0.000	0
**L19-2018**	20	H4b1	1.485	3	0.664	1
**L21-2015**	20	J1c2e1	2.883	8	1.220	2
**L21-2017**	34	N1a1a1a1	2.197	6	0.606	1
**L2-2018**	28	U4c1	1.850	6	0.000	0
**L23-2008**	31	I2e	2.261	6	0.606	1
**L24-2006**	n/a	T1a1b	2.793	7	1.239	2
**L24-2018**	47	H1	0.821	2	0.000	0
**L26-2002**	19	H2a1c	0.821	2	0.000	0
**L28-2016**	16	U5b1b1a	1.235	4	0.000	0
**L29-2005**	n/a	H3g4	0.821	2	0.000	0
**L32-2006**	30	HV6a	1.004	3	0.000	0
**L32-2016**	15	K1c1h	2.257	6	0.609	1
**L39-2008**	40	U8b1a2b	2.557	6	0.688	1
**L41-2005**	38	U5a1d1	3.060	9	0.000	0
**L4-2015**	23	K1a1b1a	2.257	6	0.609	1

**L5-2014**	56	H3v	0.452	1	0.000	0
**L7-2018**	42	B4c1b2	2.541	6	1.142	2
**L8-2019**	33	H1c	0.821	2	0.000	0
**L9-2010**	35	H7c1	0.821	2	0.000	0

**Control male subject ID**	**Age in years**	**mtDNA haplogroup**	**Total variant load**	**Number of variants**	**Variant load using variants with MutPred score >0.5**	**Number of variants with MutPred score >0.5**
**KJ2**	84	H92	0.821	2	0.000	0
**KJ6**	71	H1c+152	0.821	2	0.000	0
**KJ7**	70	H1c+152	0.821	2	0.000	0
**KJ8**	64	H18	1.230	3	0.000	0
**KJ12**	60	U5a1d2a1	2.665	7	0.601	1
**KJ13**	52	H	0.821	2	0.000	0
**KJ14**	72	H3+152	0.821	2	0.000	0
**KJ15**	71	J1c2c1	3.253	9	1.220	2
**KJ21**	76	T2a1	2.588	7	1.239	2
**KJ22**	82	H2a1c	0.821	2	0.000	0
**KJ23**	69	U5a2a1	2.019	6	0.000	0
**KJ26**	63	H+16311	0.821	2	0.000	0
**KJ31**	77	X2b4	2.349	6	0.569	1
**KJ38**	64	W1	2.719	7	0.505	1
**KJ40**	81	J1c9	2.883	8	1.220	2
**KJ45**	83	H16d	0.821	2	0.000	0
**KJ47**	85	H5r	0.821	2	0.000	0
**KJ49**	77	J1c5	2.883	8	1.220	2
**KJ52**	60	H3	0.821	2	0.000	0
**KJ55**	77	H11a1	1.072	3	0.000	0
**KJ56**	61	V1a1	2.629	6	0.594	1
**KJ61**	78	V7a	0.821	2	0.000	0
**KJ63**	87	X2e2b	1.716	5	0.000	0
**KJ66**	83	J1c2	3.509	9	1.846	3
**KJ67**	62	H5e1a1	0.821	2	0.000	0
**KJ68**	68	V1a	1.885	4	0.594	1
**KJ70**	84	HV9	0.821	2	0.000	0
**KJ75**	69	U5a2a1	2.019	6	0.000	0
**KJ76**	72	I1a1a	2.714	7	1.188	2
**KJ79**	n/a	U4a2b	1.215	4	0.000	0
**KJ104**	60	H16	0.821	2	0.000	0
**KJ106**	56	N1b1a1	3.070	7	1.275	2
**KJ112**	65	J1c2	3.509	9	1.846	3
**KJ116**	83	H1c1	0.821	2	0.000	0
**KJ117**	76	H11b1	1.581	4	0.509	1
**KJ118**	69	V7a	0.821	2	0.000	0
**KJ120**	79	H7c3	0.821	2	0.000	0
**KJ121**	61	J1c5	2.883	8	1.220	2
**KJ123**	57	H28a	1.320	3	0.000	0
**KJ129**	65	V1a1	2.629	6	0.594	1
**KJ131**	66	J2a1a1a2	3.059	8	1.396	2
**KJ153**	n/a	U8a1a1b	2.630	6	1.331	2
